# High blood flow shear stress values are associated with circulating tumor cells cluster disaggregation in a multi-channel microfluidic device

**DOI:** 10.1371/journal.pone.0245536

**Published:** 2021-01-14

**Authors:** Alessandra Marrella, Arianna Fedi, Gabriele Varani, Ivan Vaccari, Marco Fato, Giuseppe Firpo, Patrizia Guida, Nicola Aceto, Silvia Scaglione

**Affiliations:** 1 National Research Council (CNR), Institute of Electronic, Computer and Telecommunications (IEIIT), Genoa, Italy; 2 Department of Computer Science, Bioengineering, Robotics and Systems Engineering, University of Genoa, Genoa, Italy; 3 Department of Physics, University of Genoa, Genoa, Italy; 4 Department of Biomedicine, Cancer Metastasis Laboratory, University of Basel and University Hospital Basel, Basel, Switzerland; University of New South Wales, AUSTRALIA

## Abstract

Metastasis represents a dynamic succession of events involving tumor cells which disseminate through the organism via the bloodstream. Circulating tumor cells (CTCs) can flow the bloodstream as single cells or as multicellular aggregates (clusters), which present a different potential to metastasize. The effects of the bloodstream-related physical constraints, such as hemodynamic wall shear stress (WSS), on CTC clusters are still unclear. Therefore, we developed, upon theoretical and CFD modeling, a new multichannel microfluidic device able to simultaneously reproduce different WSS characterizing the human circulatory system, where to analyze the correlation between SS and CTC clusters behavior. Three physiological WSS levels (i.e. 2, 5, 20 dyn/cm^2^) were generated, reproducing values typical of capillaries, veins and arteries. As first validation, triple-negative breast cancer cells (MDA-MB-231) were injected as single CTCs showing that higher values of WSS are correlated with a decreased viability. Next, the SS-mediated disaggregation of CTC clusters was computationally investigated in a vessels-mimicking domain. Finally, CTC clusters were injected within the three different circuits and subjected to the three different WSS, revealing that increasing WSS levels are associated with a raising clusters disaggregation after 6 hours of circulation. These results suggest that our device may represent a valid in vitro tool to carry out systematic studies on the biological significance of blood flow mechanical forces and eventually to promote new strategies for anticancer therapy.

## Introduction

Cancer metastasis is a biologically complex tumor dissemination process associated with a poor survival rate. During this process, circulating tumor cells (CTCs) detach from a primary tumor and exploit the physiological blood circulation to give origin to metastases at secondary sites [1,2]. In particular, cancer cell motility enables their invasion into the blood vessels, in a phase known as intravasation, to then flow through the circulatory system up to reach localized and distant sites [3,4]. Although CTCs are present in the blood of oncologic patients, the amount of these cells is very low if compared with other cells like blood cells or leukocytes [5,6]. In particular, CTCs flow the bloodstream as single cells or as multicellular aggregates (CTC clusters), which present with a higher potential to metastasize [7]. Specifically, when transported in fluids, single CTCs and CTC clusters are subjected to various fluid-dynamic forces affecting cell death or extravasation [8].

Therefore, different studies have recently investigated the role of the bloodstream on the CTCs survival and aggressiveness to better understand the metastatic cascade and to identify more effective therapeutic approaches [9–11]. In particular, physical blood parameters, including pressure, fluid velocity and shear stress, may affect the morphology, the expression of specific biomarkers and aggressiveness of CTCs [12,13].

The tumor vasculature is capillary-based with a very low fluid flow velocity, where CTCs are subjected to mild SS (10–20 dyn/cm^2^), mostly due to the small section of the blood capillary. As they are transported far from the primary site, some CTCs flow through the arterial and venous system, where they encounter higher SS (4–30 dyn/cm^2^) and lower SS (1–4 dyn/cm^2^), respectively [8].

How individual CTCs and CTC clusters can withstand fluidical stimuli and survive in their way in the circulatory system is not yet fully elucidated. Mathematical models and computational fluid-dynamics (CFD) analysis based on physical principles can be extremely useful for investigating single CTCs and CTC clusters behavior in the vascular microenvironment. In fact, the use of algorithms opens a wide range of scenarios for modeling biological processes and optimizing the performance of device and materials conceived to interact with biological tissues [14,15]. Moreover, CFD analysis together with experimental data may allow to predict how cellular networks and systems respond to changes in their environment and therefore can help in defining which parameters affect their activity [16,17]. Indeed, a computational-experimental approach has recently elucidated the influence of cellular biophysical properties on hemodynamic deformation and margination of CTC aggregates within the bloodstream, successfully highlighting the fluid dynamics contribution during the CTCs dissemination [18,19].

In this scenario, in vitro microfluidic systems have been emerged paving the way towards novel in vitro cell-culture formats able to better reproduce the fluid-dynamical features of the cancer environment [20,21]. In fact, although in vivo models are more physiological, they still present some experimental limitations [22]. In particular, it is difficult to in vivo independently capture the effects of individual microenvironmental variables (i.e. rigidity of the ECM matrix, bio-mechanical features of the blood vessels, blood fluid-dynamical proprieties) on the development of biological processes and extract quantitative data [23–27].

The advances in microfluidic technologies enabled in vitro modelling of tumor-related biophysical factors affecting different cell types [28–30]. Moreover, it was already shown that SS is one of the prominent physical stimuli that alters the cancer cells motility and migration, the adhesion capacity and the viability and cluster activity of CTCs within the bloodstream [31–35].

However, to date, most of the systems aimed to study in vitro the effects of fluidic stimuli (such as SS) in vitro on tumor cells are based on rudimental single-channel fluidic systems connected to a peristaltic pump or systems based on needles connected to syringe pumps, where it is possible to get different SS values, by changing the flow rate at the pump level for each experimental condition [10,11,36].

In this work, we have designed and realized a novel microfluidic device able to simultaneously reproduce different hemodynamic WSS in the same experiment, thanks to a specific design based on increasing levels of vessels ramifications, finally reducing biological (experimental) variability.

To fabricate the 3D vessels interconnections, 3D printed technology was adopted to produce a 3D master with the desired fluidic pattern. Polydimethylsiloxane (PDMS) was then molded against the sacrificial 3D printed template, thus obtaining microfluidic PDMS channels, difficult to achieve through standard soft lithography.

We then examined the effects of different WSS on circulating metastatic breast cancer cells (MDA-MB-231) as single cells and as clusters of cells, respectively, by injecting a cells suspension in the microfluidic device.

This system can be further used to analyze the drug efficacy towards CTCs in circulation as well as to improve a deeper understanding of which factors allow single CTCs and CTC clusters to survive in the bloodstream, with the ultimate goal to design next generation therapies against the metastatic spread of cancer.

## Materials and methods

### Theoretical and CFD analysis

Firstly, a theoretical model, based on the Continuity law and energy balance, was developed to reproduce different shear stress values within the device. In particular, different levels of ramifications were designed in order to obtain multiple shear stress in three distinct circuits by setting a unique inlet flow rate at the level of the pump. The theoretical model is based on the hypothesis of i) laminar flow within vessels, i.e. channels, with rectangular geometry and with the same length; ii) the cells are dispersed in a medium similar to water as Newtonian fluid.

It was then possible to calculate the number of ramifications for each circuit to get the proper wall shear stress (WSS) by following (1):

$$\frac{Q^{in}}{n}=\frac{\tau BH^{2}}{6\mu }$$ (1)

where *Q*^*in*^ is the inlet flow rate at the pump level, *τ* is the WSS in the vessels, *B* and *H* the height and width of vessels, respectively, μ is the water dynamic viscosity, n the number of vessels. Thanks to this equation it is possible to design the proper number of ramifications for each circuit to obtain the desired range of shear stress within the device, by setting a unique flow rate at the pump level.

Since we wanted to reproduce the range of physiological shear stresses at the different vascular tree districts ranging from is 1–20 dyn/cm^2^, three independent set of branches were designed to reproduce the following different levels of WSS: 2-5-20 dyn/cm^2^.

The obtained geometry was designed through a commercial software. The fluid was assumed to be incompressible and in laminar flow regime, therefore the fluid dynamics within the circuit was modelled by using the Laminar Fluid Flow module of Comsol Multiphysics 5.5. The equations solved are the Navier-Stokes ones for conservation of momentum (2) and the continuity one for conservation of mass (3):

$$\rho \left(\frac{\partial u}{\partial t}+u∙\nabla u\right)=-\nabla p+\mu \nabla^{2}u$$ (2)

ρ(∇∙u)=0 (3)

where *u* is the velocity in the vessels and *p* the pressure across the circuit. The density ρ and dynamic viscosity μ values were specified for water at room temperature (25°C).

As initial conditions, the velocity field and the pressure were considered null.

No-slip boundary condition was fixed because there is no flow across the device walls.

An inflow laminar rate equal to 30 ml/min was set as inlet for each of the three circuits, whereas an atmospheric pressure condition with no backflow was chosen as outlet.

Since the steady state flow is reached almost instantaneously (~0.005 s to ~0.01 seconds) for flow rates range between 24–36 ml/min, we considered only a steady state analysis avoiding transient laminar flow simulations.

An iterative geometric multigrid (GMRES) algorithm was used to solve the equations for the steady-state condition.

### Microfluidic device fabrication

#### 3D printing of the masters

The device negative masters were designed through the Computer-Aided Drafting (CAD) software Sketchup 2016, accordingly with the theoretical and CFD analysis described above. The 3D masters were printed with a biocompatible photopolymer resin (Dental LT Resin) by using a 3D-printer (Form2, Formlabs) with a resolution of 100 μm.

After printing, the masters were washed with isopropyl alcohol for 10 minutes and then photo-cured through UV light for 20 minutes in order to eliminate resin residues and to optimize the photo-crosslinking process, respectively.

Then, a silanization treatment was performed to facilitate the successive rapid removal of PDMS replicas from the masters during the replica molding (REM) phase [15]. Briefly, it consists in an oxygen plasma treatment for 240 seconds with 50 W (Tucano plasma reactor Gambetti Kenologia) and in the vapor phase deposition (p = 30 kPa) for 30 minutes of trichloro(1H,1H,2H,2H-perfluorooctyl)silane (FOTS) antiadhesive agent (Sigma-Aldrich).

#### PDMS molding

Positive replicas were fabricated in PDMS (Sylgard® 184 Silicone Elastomer Kit, Dow Corning) by curing the pre-polymer (a mixture of 10/1 w/w base and the curing agent) on the printed masters at 60°C for 2 hours, accordingly with the previously described protocol [16]. The PDMS mold was removed from the printed master by manually peeling it off (demolding). This procedure resulted in replicas with intruded and top-opened channels with rectangular cross-sectional geometry, as a result of the negative 3D-printed master. The patterned replica was bonded to a PDMS flat layer (i.e. a closing layer of 175 mm x 70 mm x 1 mm in size), that was previously fabricated by casting and curing PDMS pre-polymer in a proper plastic master fabricated by standard stereolithography. Both masters were then reused without additional cleaning processes.

Once demolded, both the fabricated PDMS layers were irreversibly sealed together, resulting in a final polymer device. In details, the sealing procedure is the following. Firstly, a pre-polymer film was deposited directly on a 200 mm x 200 mm glass plate, with a thickness lower than 50 μm. Then, the patterned replica and coated glass were arranged to allow a fast inking contact (5 minutes) between the open-side of the replica’s channels and the film. Successively, the replica was manually detached from the coated glass and exposed to partial curing at 60°C for no more than 5–8 minutes, obtaining a sticky side. Then, the sticky layer was placed in contact with the closing layer and bonded together by curing in oven at 60°C for 30 minutes. Finally, the plastic tubes of the circuit were connected to the holes at the extremities of the channel and permanently sealed by applying the pre-polymer at the interfaces, and by curing it at 60 ˚C for 30 minutes to fix the connection.

#### Hydrophilic treatments

To make a hydrophilic surface layer on PDMS, the native PDMS surfaces were modified by oxidation. The oxidation process was firstly performed by plasma oxidation, placing the assembled PDMS device in the oxygen plasma chamber for 120 seconds with 50 W [17], and then by exposure to an oxidizing solution of H2O:HCl:H2O2 (in a volume ratio of 1:1:5) [20]. The PDMS channels were filled with this solution and maintained for 10 minutes at 70°C, followed by two washes in DI-water, thus resulting in a hydrophilic silanol-covered (Si-OH) PDMS surface.

Since in contact with air the PDMS surface reverts to hydrophobic, the oxidized channels’ surfaces were treated with a silanization solution of 5% 3-aminopropyltriethoxysilane (APTES, Sigma-Aldrich) for 20 minutes at 80°C, followed by an EtOH rinse to remove the unreacted APTES. The fluidic device was then baked at 80°C for 30 minutes in an oven, followed by rinsing with a 1X-PBS (ThermoFisher Scientific) solution for 5 minutes. After this procedure, the channels’ surface results in aamino-grafted PDMS and glass-like surfaces that remain stable over time [21].

### Morphological analysis of the device

The PDMS patterned replicas were analyzed under an upright epifluorescence microscope (BX51, Olympus) to detect that no cracks were formed along channels during the PDMS removal process. The optically transparent PDMS device enabled independent channel defect or leakage verification using dispensed a dye consisted of 1% methylene blue (Alfa Aesar) in deionized water.

### Metastatic breast cancer cells circulation

The device was sterilized in ethanol 70% v/v for 30 minutes followed by rinsing with sterile DI water and connected to a syringe pump (Harvard apparatus PHD2000) using connectors (Biorad) and Tygon tubes (i.d = 1 mm). To reduce the cell adhesion to the inner surface of the tubes, these were treated with 1% Pluronic F-127 (Sigma Aldrich) for 30 minutes. MDA-MB-231 cells (from breast adenocarcinoma) were expanded in Roswell Park Memorial Institute medium (RPMI) enriched with 10% Fetal Bovine Serum (FBS), 1% L-glutamine and 1% penicillin/streptomycin (all from Sigma Aldrich). The culture media was changed twice a week. At confluence, cells were enzymatically detached with 0.05% trypsin, counted and injected within the three different circuits, with a 21G needle, at a density of 2 x 10^5^ cells/ml. The syringe pump flow rate was set at 30 ml/min to reproduce the different WSS values. Cells cultured over non-adhesive surfaces were used as static control.

### Cell viability analysis of recovered cells

MDA-MB-231 viability after circulation within the device was evaluated through a live/dead assay (Sigma Aldrich). Briefly, after 6 hours of culture the same volume of cells from different circuits was placed in 96-well plates. Cells were allowed to adhere for 24 hours, were washed with PBS and incubated in 2 mM calcein AM and in 4 mM EthD-1 in PBS for 15 minutes at 37°C in a dark environment, to detect live and dead cells, respectively. Cells were washed three times in PBS. Positivity to either staining solution was observed by means of fluorescence microscopy (Nikon H550L). Viable cells were counted by using Image J® software from binarized images. In particular, cells positive to calcein were assessed through image post-processing of binarized images, by calculating the number of cells present in the green channel.

### CFD analysis of cell clusters disaggregation

The hemodynamic disaggregation of CTC clusters due to the blood flow-associated forces was simulated, by considering three different physiological WSS values (2-5-20 dyn/cm^2^).

Each cluster circulating in the vascular system was assumed as a fluid with physical properties, such as density, dynamic viscosity, etc., equal to those typical of the clusters. In particular, the values were set considering the spatial average values of the cluster.

Thus, the clusters motion and disaggregation within the blood vessels were simulated by considering the flow of two different immiscible fluids, one representing the blood and the other one representing the clusters. Accordingly, a two-phase flow model was set up by combining the Laminar Fluid Flow module with the Level Set one of Comsol Multiphysics 5.5, following a computational approach recently proposed and adopted by Phillips and colleagues [37].

In fact, the level-set method was used to track moving interfaces in fluid flows models taking into account differences in fluids densities and viscosities, and also including the effects of surface tension, by solving the transport equation for the level set function *ϕ* (4):

$$\frac{\partial \phi }{\partial t}+u∙\nabla \phi =\gamma \nabla ∙\left(\varepsilon \nabla \phi -\phi (1-\phi )\frac{\nabla \phi }{\left|\nabla \phi \right|}\right)$$ (4)

where *γ* is a reinitialization parameter, set to the maximum expected velocity magnitude, and *ε* the interface thickness controlling parameter, set to h_max_/2 where h_max_ is the maximum element size in the component.

Since the velocity field is not divergent, a conservative level set method was adopted because it perfectly conserves the mass of each fluid, even if the computational time is longer.

Moreover, in most level-set methods the right-hand side of (4) is zero, but in the current method it was necessarily filled to keep numerical stability and interface thickness [38].

The auxiliary function *ϕ* (5) varies smoothly from 0 (fluid 1) to 1 (fluid 2), passing through the interface where it is equal to 0.5.

$$\phi =\left\{ \begin{array}{c} <0.5\;water\;(fluid\;1)\\ 0.5\;interface\\ >0.5\;cluster\;(fluid\;2) \end{array}\right.$$ (5)

Finally, the incompressible formulation of the Navier-Stokes equation solved for the laminar flow regime becomes:

$$\rho \left(\frac{\partial u}{\partial t}+u∙\nabla u\right)=\nabla ∙[-pI+\mu (\nabla u+\nabla u^{T})]+F_{st},$$ (6)

where *ρ* is the density, *μ* the dynamic viscosity and *F*_*st*_ the surface tension force, which allows the model to incorporate the cluster deformability, defined as:

$$F_{st}=\sigma \delta kn+\delta \nabla_{s}\sigma ,$$ (7)

where *σ* is the surface tension coefficient, *n* the normal to the interface and *k* the curvature:

k=−∇∙n, (8)

*δ* a Dirac delta function located at the interface and *∇*_*s*_ the surface gradient operator:

$$\nabla_{s}=(I-nn^{T})\nabla$$ (9)

The density is a function of *ρ*_1_ (1000 kg/m3) and *ρ*_2_ (1500 kg/m^3^) [39], densities of fluid 1 and fluid 2 respectively, defined as:

$$\rho =\rho_{1}+(\rho_{2}-\rho_{1})\phi ,$$ (10)

whereas, the dynamic viscosity, also depending on *ϕ*, is a function of *μ*_1_ (0.001 Pa∙s) and *μ*_2_ (1.36 Pa∙s) [37], dynamic viscosities of fluid 1 and fluid 2 respectively, given by:

$$\mu =\mu_{1}+(\mu_{2}-\mu_{1})\phi .$$ (11)

As initial conditions the velocity field and the pressure of each fluid were considered null, whereas a no-slip condition for boundaries was fixed.

The computational domain was designed to reproduce a single device channel characterized by rectangular shape with 1 mm of width and 120 mm in length. To avoid high computational timing, the geometry was implemented in two dimensions.

In order to obtain the three different levels of WSS within the channels (2-5-20 dyn/cm^2^), at inlet an inflow laminar rate was set to 2-5-20 ml/min respectively (1).

A user-defined unstructured triangular mesh was used. In particular, an interface refinement and a boundary layers elements densification were performed by splitting them.

Finally, in order to automatically initialize the level-set variable, a Phase Initialization study was performed and then a Time Dependent step was implemented, since the position of an interface always depends on its history.

### Experimental analysis of cell clusters disaggregation

Clusters of MDA-MB-231 were generated by detaching 80%-confluent monolayer of cells with trypsin for 1 minute. Clustered MDA-MB-231 were then counted and injected within the three different circuits, with a 21 G needle, at a density of 1 x 10^3^ clusters/ml. The same needle was used to add the clusters suspension in a non-adhesive 96-well plate as static control. The same procedure was also performed to remove the clusters from the device and the non-adhesive static control after 6 hours of circulation.

Samples were then observed under the microscope. Images obtained by an optical microscopy were analyzed through ImageJ® software, to quantify morphological features of the clusters after circulation. In particular, the following morphological parameters were measured: cluster size, defined as the diameter of the cluster calculated as the average between the major and minor axis length; cluster area; circularity, defined as 4πarea/perimeter^2^; and perimeter. Quantification of the clusters diameter, area, circularity and perimeter was performed with ImageJ® on thresholded images through a semiautomatic image postprocessing of binarized images. Within each experiment, the same threshold was used for all the conditions.

## Results

### Theoretical and CFD analysis

Three distinct circuits composed by ramified vessels have been designed within the device to simultaneously and independently reproduce three different levels of WSS, resembling the human vascular system.

**Fig 1:**
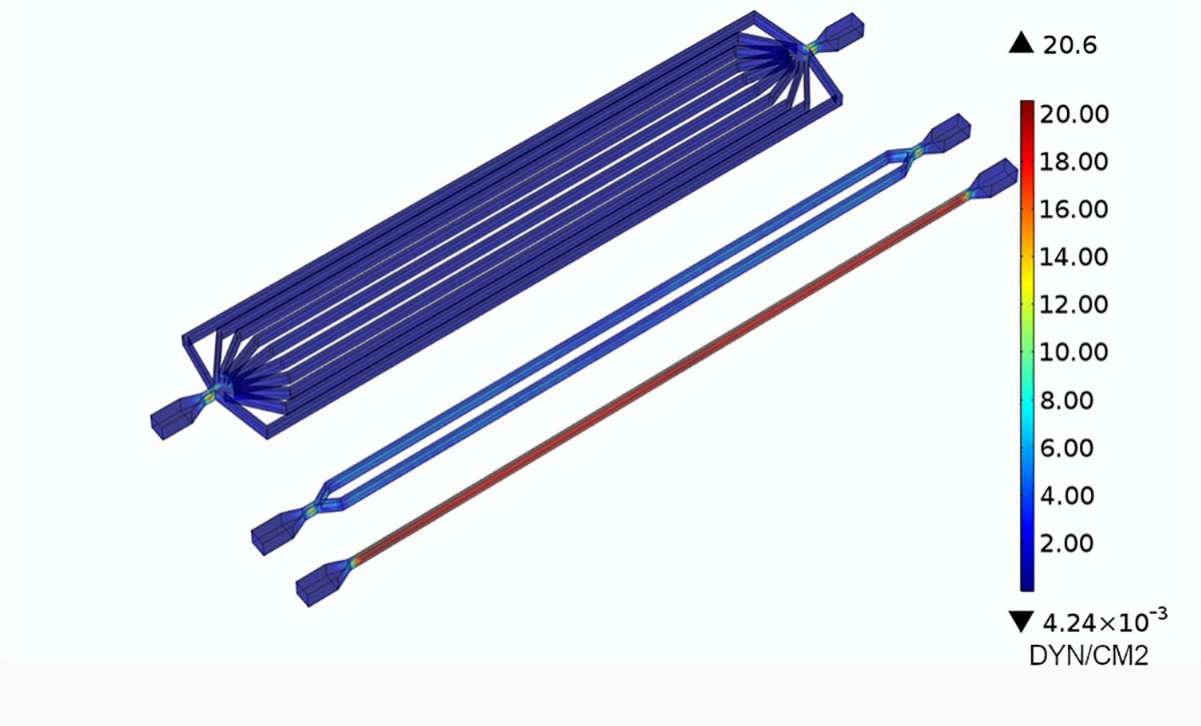
SS profiles of the fluid flows. SS profiles of the fluid flow within the channels with an inflow rate of 30 ml/min.

Based on the theoretical mathematical model, the number of designed ramifications is 10 for the first circuit, 2 for the second circuit, and 1 for the third one (Fig 1), to obtain with a unique flow rate the desired physiological WSS values of 2, 5, 20 dyn/cm^2^, corresponding to those present in veins, arteries and capillaries.

To predict the fluid dynamics within the circuits, a computational analysis of the fluid dynamics within the device was performed, and the WSS values within the microfluidic vessels evaluated.

Fig 1 reports the different WSS values at the three levels of ramifications. The figure shows that the desired values of WSS are obtained by setting at inlet the same flow rate at 30 ml/min, as anticipated by the theoretical model.

Table 1 reports the shear stress in the microfluidic vessels for the flow rate tested in this work. The shear stress values obtained from the simulations are close to the ones predicted with the theoretical model, showing the good reliability of the model.

**Table 1:** Wall Shear stress within three different ramifications.

N of ramifications	WSS [dyn/cm^2^]
1	20±0.03
2	5±0.02
10	2±0.18

Wall shear stress within three different ramifications when a 30ml/min flow rate was applied.

### Device fabrication

The fluidic system was composed of different parts: the PDMS micro-channel device; plastic tubes physically connected to the device which allows cells circulation; a syringe pump which drives the fluid flow.

To simultaneously reproduce different hemodynamic WSS on a single device, the microfluidic device developed in this study comprises three independent circuits composed of ramifications of channels connected respectively to three inlets and three outlets.

The specific pattern of three independent circuits—composed of ramifications of channels connected respectively to three inlets and three outlets was designed according to the theoretical and CFD analysis.

**Fig 2:**
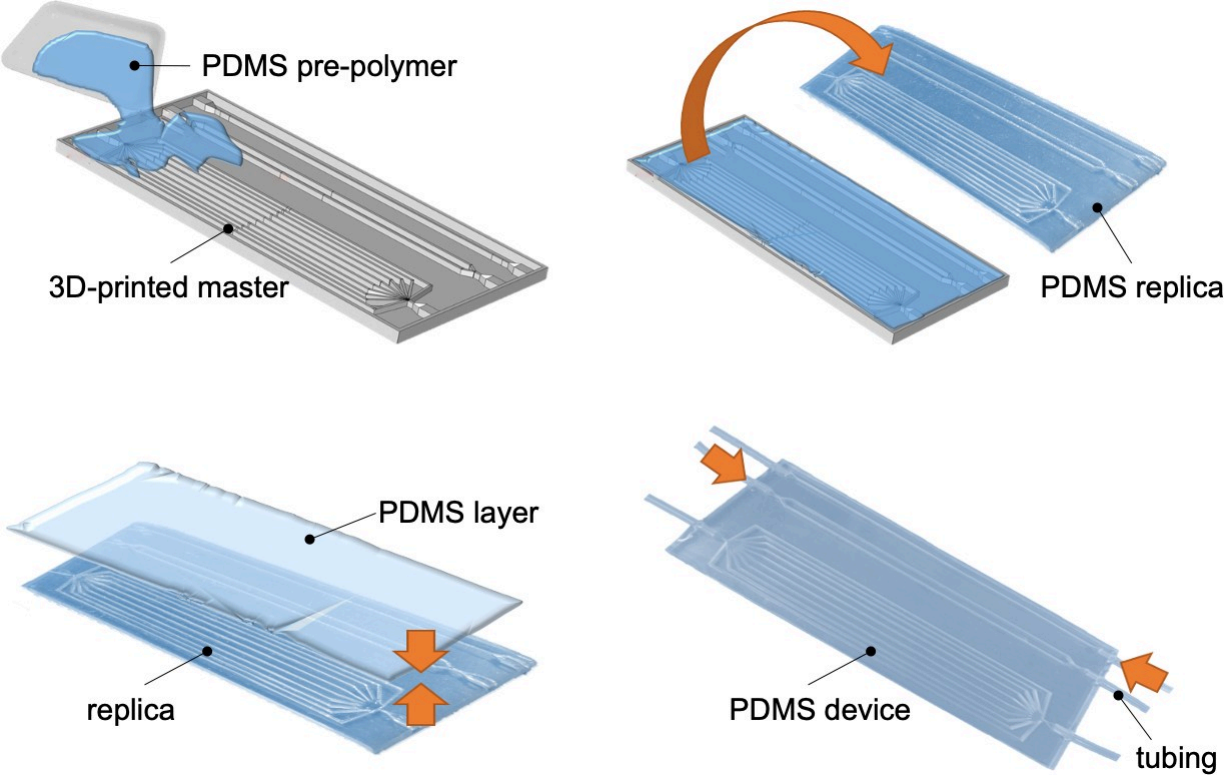
PDMS device fabrication process. Schematic representation of the fabrication process of the device.

We utilized a procedure based on 3D-printing and soft-lithography techniques to fabricate the flow circuits in PDMS (Fig 2), ensuring a well-defined size and shape of channels with optical transparency (down to 280 nm).

In particular, after the designing step, the channels pattern was converted into 3D-printing code and printed under precise digital control as a solid polymeric three-dimensional master (Fig 2). The master contained extruded tube-shaped structures of rectangular cross-section with 1 mm × 3 mm in size and 120 mm in length. The device was then realized in PDMS by curing the pre-polymer on the printed template, then the PDMS mold was removed from the printed masters by manually peeling it off.

**Fig 3:**
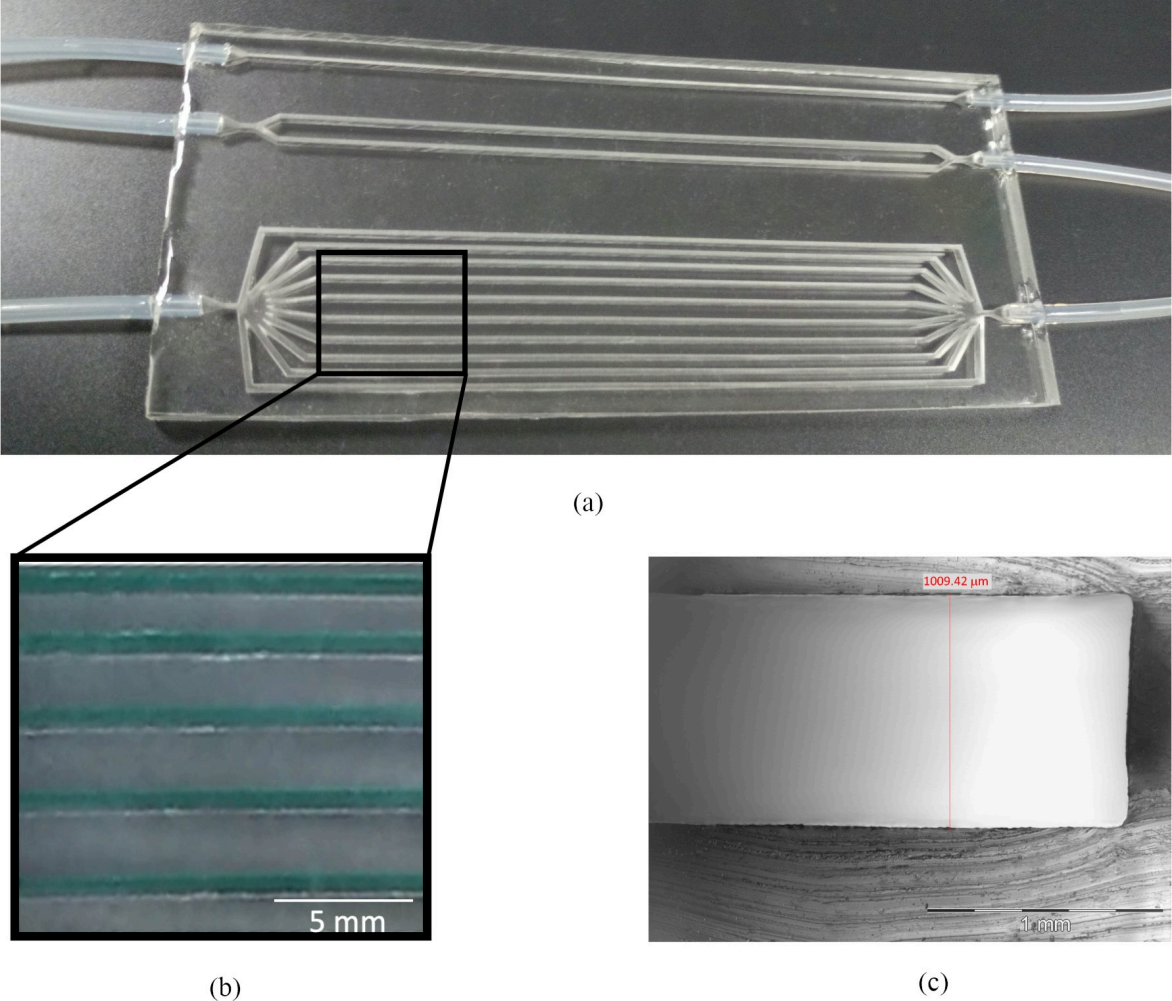
PDMS multi-channel device. Pictures showing (a) the 3D fluidic PDMS device with different ramifications of channels, inlets/outlets are permanently connected to tubes, (b) the same channels filled with a blue dye to demonstrate absence of leakage and (c) the cross-section of a PDMS channel.

After completing this reliable manufacture process, a fluidic device (175 mm × 70 mm × 4.5 mm) was realized in PDMS (Fig 3A), to guarantee low cost, biocompatibility, gas-permeability and robustness. In particular, the channels were deep 3 mm to avoid their collapse during fabrication.

Before and after sealing, we analyzed the integrity of the channels by injecting methylene blue and observing under an upright epifluorescence microscope. No entrance of methylene blue dye was observed into the walls of channels and the seal areas (Fig 3B). A cross-section of the channels made in PDMS after removal from the mold was evaluated (Fig 3C) and show the successful realization of 1mm wide channels.

### Cell viability

MDA-MB-231 cells were cultured within the fluidic device to validate the system and analyze the effects of WSS on CTC viability. After 6 hours of circulation within the device, the cells were collected and re-plated in multi-well plates. Image post processing was performed to quantify the number of viable cells. Cell viability was inferred from the number of green (viable) cells for each sample, respectively to the number of viable cells in the static control.

**Fig 4:**
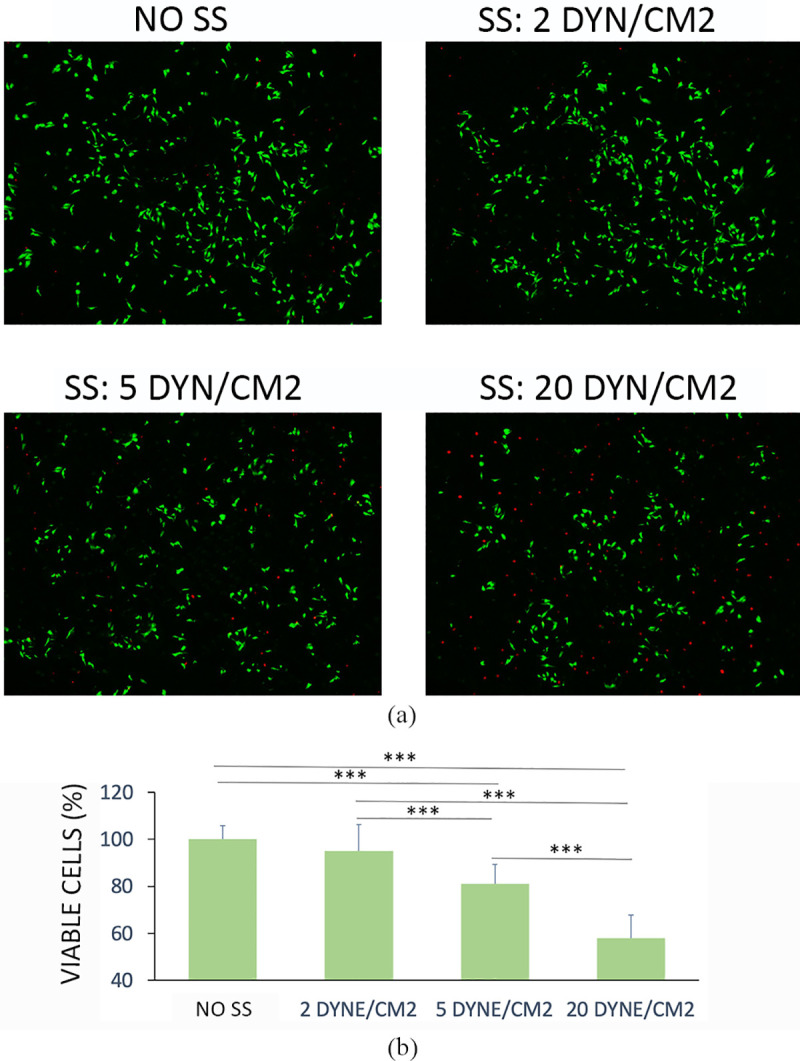
Cell viability. (a) Cell viability measured by live/dead staining of cells replated after 6 hours of circulation (b) Quantitative analysis of the images. Values are reported as mean ± s.d., N = 4. Student's t-test. ***P < 0.001.

The fluorescent micrographs showed that a low WSS (2 dyn/cm^2^) didn’t significantly affect cell viability, being most of the cells alive and thus able to adhere unto plastic surfaces, compared to the static control. Moreover, results showed that high WSS affected cell survival: in particular, after 6 hours of circulation at 20 dyn/cm^2^ the cells recovered and adhered in multiwells were less in number, as effect of a reduced viability (Fig 4).

### Cell clusters CFD simulations

To reproduce CTC clusters disaggregation, depending on the WSS level experienced by the clustered cells, mathematical simulations were performed in a two-dimensional (2D) domain mimicking a single micro-channel of the device.

Therefore, three different physiological-relevant WSS conditions (2, 5, 20 dyn/cm^2^) were investigated. Since each cluster was represented by a fluid, we implemented a two-phase flow model by using Comsol Multiphysics 5.5.

**Fig 5:**
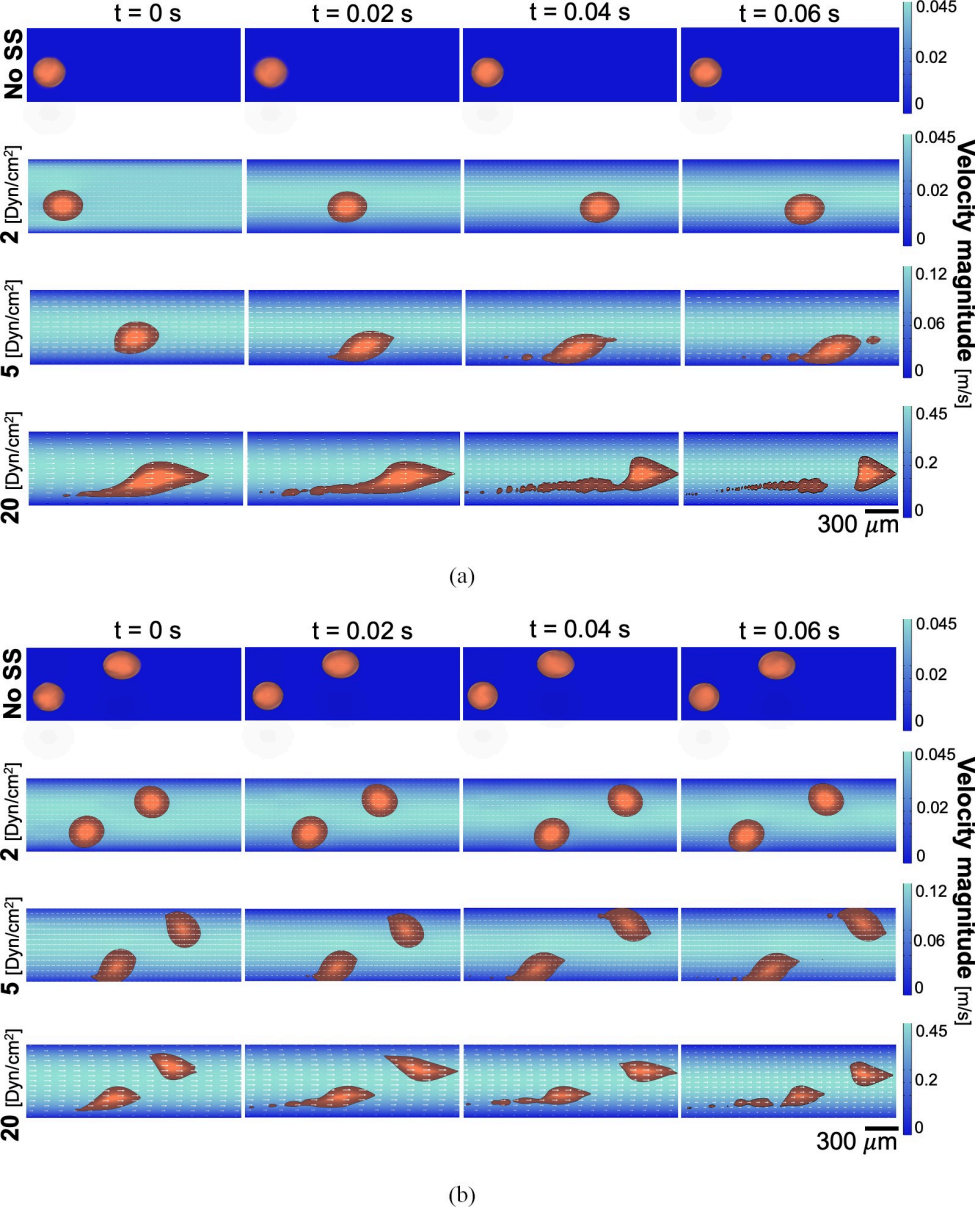
CTC clusters disaggregation. Fluid-dynamic simulation of CTC clusters disaggregation process under fluid flow with (A) one and (B) two clusters circulating. Clusters shape inside the vessel is reported at different time points: t = 0, 0.02, 0.04 and 0.06 s.

Fig 5 shows the different SS effects on CTC clusters. In particular, based on the simulations, increasing SS levels should induce a major cluster disaggregation. Under a WSS equal to 20 dyn/cm^2^, the clusters disaggregation gives origin to bigger newly derived clusters, thus highly reducing their original size; while when the WSS is lower (5 dyn/cm^2^), smaller derived aggregates are observed. Moreover, no significant differences can be noticed between the 2 dyn/cm^2^ condition and the control one (no SS).

Furthermore, it can be observed how the clusters align along the flow direction to decrease their hydrodynamic resistance in the first instants of experiment simulated (Fig 5). In addition, such mechanism is more evident as SS raises. In a recent study, this process was experimentally observed in human CTC clusters in xeno-transplanted zebrafish, where CTC clusters reduced their flow resistance by reorganizing into single-file chain-like clusters [40].

### Cell clusters experimental analysis

The here proposed fluidic device was adopted also to culture tumor cell clusters, for resembling the in vivo pathological conditions. To this aim, the role of WSS on cluster disaggregation was also experimentally investigated.

**Fig 6:**
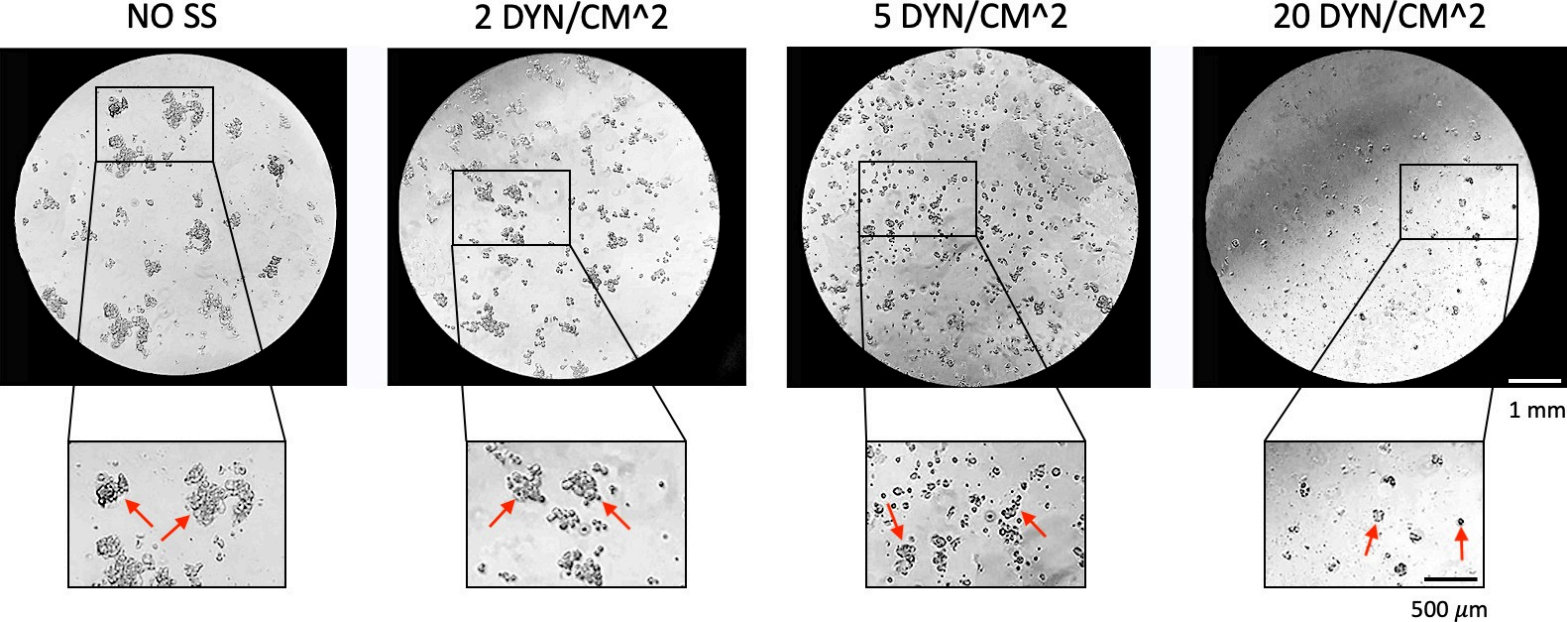
Clusters size reduction at different shear stress levels. Clusters size reduction after 6 hours of circulation at different shear stress.

Higher WSS levels increased the disaggregation of cell clusters recovered after 6 hours of circulation, as predicted by the CFD analysis (Fig 6). At low WSS (2 dyn/cm^2^) a poor reduction of cluster size was observed. At high WSS (20 dyn/cm^2^) very few and small clusters were observed and recovered.

**Fig 7:**
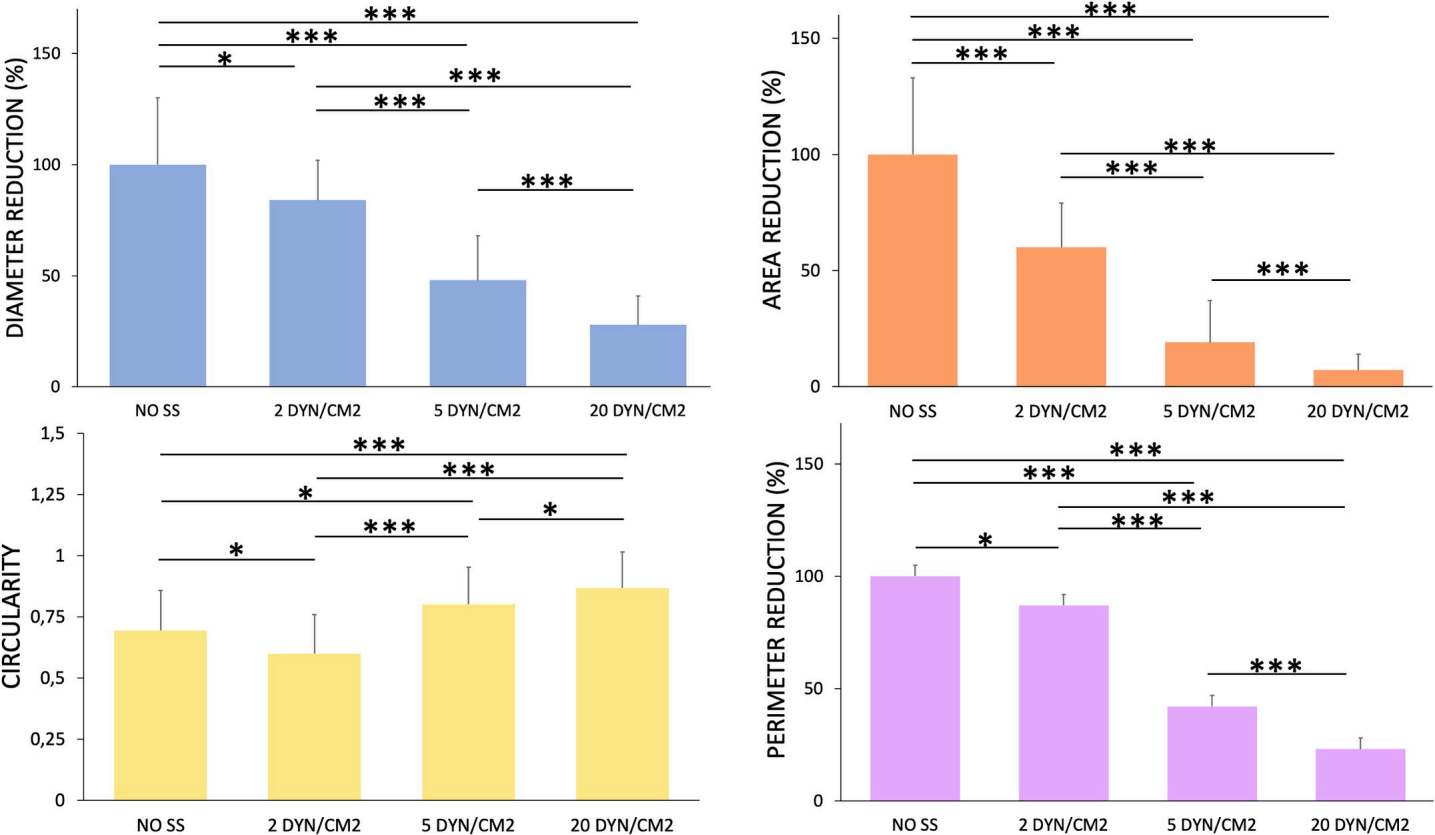
Quantitative analysis of the CTC clusters images. Values are reported as mean ± s.d., N = 4. Student's t-test. * P<0.05; ***P < 0.001.

Clusters size, area, perimeter and circularity after circulation at different SS were quantified through image post processing (Fig 7). It was found that after 6 hours of circulation at 20 dyn/cm^2^ there was a significant reduction of the clusters size up to reach a reduction of about 70% respect to the static control. Moreover, the clusters area and perimeter decrease by incrementing the values of SS confirming that higher values of SS were able to increasingly disaggregate the CTC clusters.

Interestingly, it can be observed a reduction of circularity after 6 hours of culture for 2 dyn/cm^2^, indicating a kind of cluster elongation for low values of WSS, while smaller clusters (WSS>2 dyn/cm^2^) were characterized by a higher circularity and rounded shape.

## Discussion

In the present work a micro-fluidic device able to simultaneously reproduce different physiological SS was designed and fabricated. During the first phase, theoretical and CFD models were respectively implemented. They allowed the design of three independent circuits composed by a set number of ramified channels. The number of ramifications at each entry was determined to obtain within the same fluidic device the SS values reached in vivo in capillaries, veins and arteries that together form the blood vessel circulation feeding tissues and organs. In the different anatomical sites of the vascular tree (i.e. capillaries, veins, arteries) shear stress are deeply different, reaching the highest values both in arteries, mostly due to the high flow velocities values close to 100–500 mm/s (8), and in capillaries where blood velocities are 3 orders of magnitude less but the vessel geometrical dimensions are significantly smaller. Therefore, decoupling *in vivo* the distinctive role of shear stress and velocities on CTCs behavior is still an open issue. For these reasons, we here have developed an alternative *in vitro* approach aimed to properly tune the number of ramifications for reducing the velocities differences in the three independent circuits, that are within the veins range (10–200 mm/s), and better understanding the role of the shear stresses.

The technique adopted for the device fabrication is 3D printing combined with soft-lithography. Although both 3D-printing and the PDMS-molding procedures require individual skill and expertise, they were combined to boost the respective advantages offered by 3D-printing in terms of time efficiency and complexity of the models and by PDMS-molding, due to the superior physico-mechanical properties of PDMS as substrate for microfluidic applications [41]. The fluidic device here fabricated is optically transparent and sufficiently thick to sustain pressures necessary to perform dynamic experiments and, at the same time, to allow microscopic observation.

For this application, it was also necessary to make each PDMS channel hydrophilic. since the cell culture medium should easily be injected and flow within the channels. This is challenging, due to the fast hydrophobic recovery of the PDMS surface after modification [42]. We used plasma treatment combined with the exposure to an oxidizing solution, and a coating with APTES aminosilane compound, to provide a hydrophilic functional film covalently bonded to the PDMS surfaces. In fact, such treatment reduces the nonselective adsorption of hydrophobic molecules, which is a typical limit of the hydrophobic nature of PDMS [43]. Among various methods, we opted for this one since it is suitable for a fully bonded fluidic device and ensures hydrophilicity over an extended period of time.

As first validation, we tested the viability of CTCs within the device, subjected to the different WSS reproduced.

The results obtained are consistent with previous studies reporting a decreasing cell viability along with increased WSS values of stimulation [11,36]. In particular, it was previously shown that MDA-MB-231 cells viability was about 85% and 60% after 4 hours of stimulation with WSS of 15 and 30 dyn/cm^2^, respectively [11]. In this context, the molecular pathway of WSS-mediated apoptosis was clarified by Hope et *al*. [44]. In particular, they identified mechanosensitive ion channels called Piezo1 as key players in WSS-induced TRAIL-mediated apoptosis of cancer cells (COLO 205 and MDA-MB-231 cell lines), decoding one of the possible WSS-induced mechanism impairing CTCs survival in the bloodstream.

Interestingly, in a further study it was also demonstrated that if most of highly aggressive metastatic breast cancer cells were resistant to physiological WSS (15 and 30 dyn/cm^2^), these stimuli could kill non-metastatic breast cancer cells (MCF7) by inducing apoptosis [36].

Moreover, the fluidical stimuli have a role also in the survived CTCs extravasation potential, even if the exact mechanisms are still not fully understood [32,45]. In particular, a recent work reported that cancer cell migration efficacy was 3-fold higher after a fluidic stimulation with a WSS of 15 dyn/cm^2^ if compared to a static control group [32].

Along this line, WSS around 15 dyn/cm^2^ seemed to promote also metastatic cells adhesion to extracellular matrix and endothelial monolayer. This means that WSS also conditions the effective adhesion of the extravasated tumor cells to a secondary organ with a pro-metastatic effect [33].

Therefore, these studies together with our work suggest a dual role of WSS on CTCs: if it has the capability to kill CTCs in circulation, it is also able to modulate the aggressiveness of the survived cells, fostering their capability to migrate, extravasate and adhere to a metastatic site.

Other studies showed the importance of increasing the level of complexity of the model. In fact, in a recent work authors examined the fluid WSS effects on CTC clusters by realizing a spheroid-based 3D mono and co-culture model formed by prostate cancer cells and cancer associated fibroblasts (CAFs), respectively. They demonstrated that CAFs play a pivotal role in promoting CTC survival and migration during the vascular transport, conferring shear resistance to CTC clusters through heterotypic cell-cell interaction [46].

However, despite recent advances in the understanding of biological features of CTCs, the effects of physiological WSS levels on single and clustered CTCs remain to be investigated.

**Fig 8:**
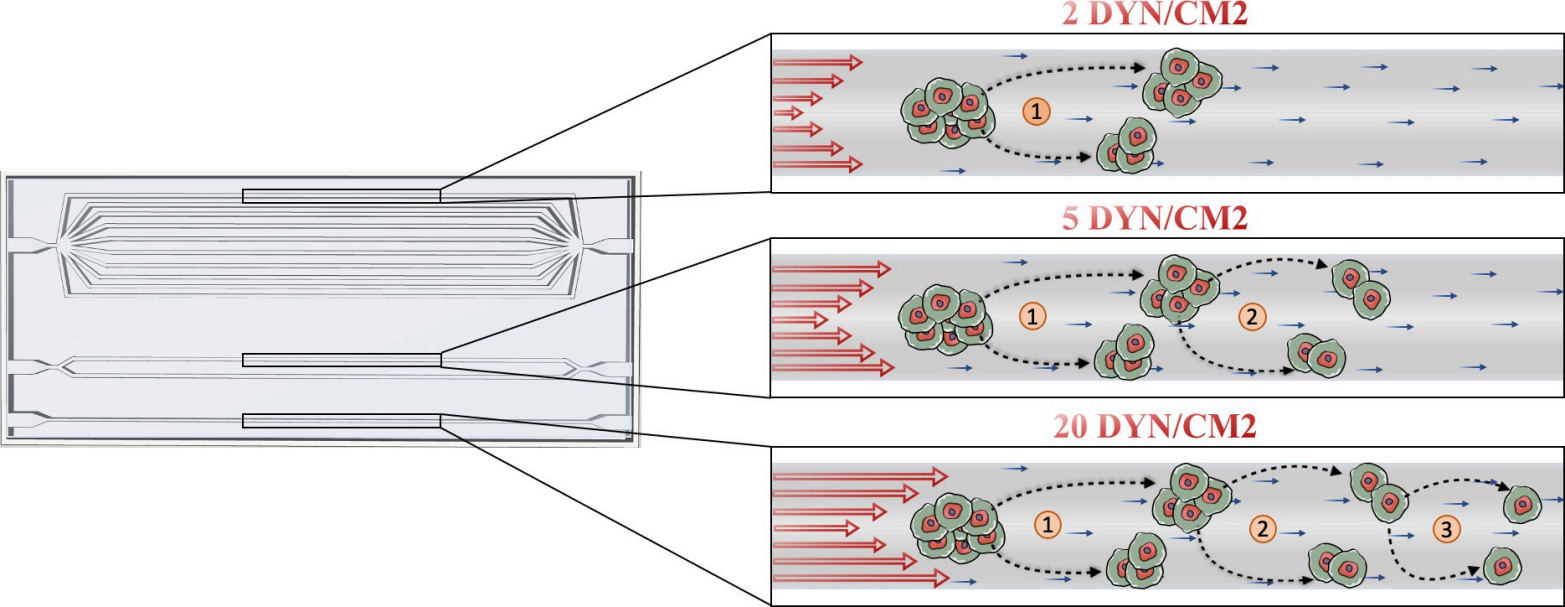
SS induce CTC clusters disaggregation. Schematic representation of the observed effects of increasing levels of SS on the disaggregation of CTC clusters.

In this context, our device may represent a valid system to study the hydrodynamical effects of the blood flow on human single or aggregated CTCs (Fig 8) and where also different co-cultures or cells derived from metastatic patients can be incorporated and analyzed.

Furthermore, CFD simulations, based on the laminar two-phase modelling, were coupled to an experimental approach to investigate the hemodynamic disaggregation of CTC clusters in response to different physiologically relevant WSS within the vessels.

In the past years, the CTCs and CTC clusters dynamics within the vasculature was modeled with other mathematical models, for example by adopting the analytical Green’s function formulation [47], where CTCs are designed as non-deformable particles, or by using the immersed finite element method (IFEM) [18], which takes into account the Young’s module of the CTC. However, in this works CTCs were modeled as spheres composed of a simple linear-elastic material and the CTC clusters as aggregations of spheres with a non-deformable structure. Therefore, it was not possible to evaluate the possible CTC clusters morphology changes that can be induced by the blood flow-associated stimuli within the bloodstream, such as the shape reorganization into single-file chain-like geometries that substantially reduce the clusters hydrodynamic resistance [40], by using this computational approach.

Hence, in our work, we employed the laminar two-phase modelling, provided by Comsol Multiphysics, following the approach proposed by a recent paper which adopted this method to in silico study the CTC clusters circulation and deformability within the vasculature [37]. In particular, each CTC cluster circulating was assumed as a fluid immiscible with the primary one (i.e. blood) with physical properties, such as density and dynamic viscosity, known from the literature [3,37,39]. Importantly, the two-phase model allowed to take into account the CTC cluster membrane deformability by considering a surface tension at the interface between the two fluids and thus to investigate the CTC cluster deformation, reorganization and disintegration in response to different physiologically relevant WSS.

Here, the results suggest that in our model the WSS has a role on the disaggregation of CTC clusters, as resumed in Fig 5 and experimentally shown in Fig 6. Specifically, MDA-MB-231 clusters were injected within the three independent circuits and their morphology after 6 hours of dynamic culture was observed.

In particular, while the clusters size decreases by increasing the values of WSS, an increased circularity of the clusters was observed. This observation can suggest that the cells within the aggregates were subjected to a kind of elongation aligning in the direction of the flow at low WSS, to then disaggregate at higher WSS values assuming the original more rounded conformation.

The capability of clusters to modulate their morphology in transit has already been demonstrated [40]. It was shown that clusters act as individual cells in series, which elongate to pass through capillary-size vessels, to then reform into the typical “rounded” organization.

Accordingly, from our results it can be hypothesized that at low WSS values (2 dyn/cm^2^) CTC clusters undergo reorganization forming chain-like structures in order to escape to the blood flow-associated forces avoiding the disaggregation process. On contrary, at higher WSS (> 5 dyn/cm^2^) cluster cells disaggregate by assuming their original rounded morphology.

## Conclusions

Metastasis is responsible of a high percentage of cancer deaths every year. The fluidic stimuli involved in this process still need to be deeply investigated. Therefore, we realized a novel microfluidic device properly designed upon theoretical and CFD modeling to simultaneously reproduce three different WSS values typical of the circulatory system.

Once validated, the effect of increasing levels of WSS on triple-negative breast cancer cells clusters was investigated through in silico and experimental studies, showing that increasing WSS values are associated with morphological variations and clusters disaggregation after 6 hours of circulation by using a breast cancer cell line.

These results show that our device may represent an in vitro system where to study the still unexplored effects of blood flow-associated physical parameters on the process of cancer metastasis, with the final goal to design and test new therapeutic strategies.
